# Mathematical Modeling Reveals the Role of Hypoxia in the Promotion of Human Mesenchymal Stem Cell Long-Term Expansion

**DOI:** 10.1155/2018/9283432

**Published:** 2018-05-14

**Authors:** Shuhua Gao, Cheng Xiang, Kairong Qin, Changkai Sun

**Affiliations:** ^1^Department of Electrical and Computer Engineering, National University of Singapore, Singapore; ^2^School of Optoelectronic Engineering and Instrumentation Science, Dalian University of Technology, Dalian, China; ^3^Research Center for the Control Engineering of Translational Precision Medicine, Dalian University of Technology, Dalian, China; ^4^School of Biomedical Engineering, Faculty of Electronic Information and Electrical Engineering, Dalian University of Technology, Dalian, China; ^5^State Key Laboratory of Fine Chemicals, Dalian R&D Center for Stem Cell and Tissue Engineering, Dalian University of Technology, Dalian, China; ^6^Liaoning Provincial Key Laboratory of Cerebral Diseases, Institute for Brain Disorders, Dalian Medical University, Dalian, China

## Abstract

Many experimental studies have found that human mesenchymal stem cells (MSCs) in long-term culture exhibited enhanced cell proliferation and prolonged lifespan under hypoxia (around 1%–7% oxygen) against the normoxic condition (about 21% oxygen). Inspired by the experimental findings, we aimed to investigate the hypoxic effects on MSC expansion quantitatively through mathematical modeling to elucidate the corresponding biological mechanism. A two-compartment model based on ordinary differential equations (ODEs), which incorporate cellular division and senescence via state transition, was developed to describe the MSC expansion process. Parameters of this model were fitted to experimental data and used to interpret the different proliferative capacities of MSCs under hypoxia and normoxia along with model sensitivity analysis. The proposed model was tested on data from two separate experimental studies, and it could reproduce the observed growth characteristics in both conditions. Overall, this compartmental model with a logistic state transition rate was sufficient to explain the experimental findings and highlighted the promotive role of hypoxia in MSC proliferation. This in silico study suggests that hypoxia can enhance MSC long-term expansion mainly by delaying replicative senescence, which is indicated by the slowdown of the state transition rate in our model. Therefore, this explanatory model may provide theoretical proof for the experimentally observed MSC growth superiority under hypoxia and has the potential to further optimize MSC culture protocols for regenerative medicine applications.

## 1. Introduction

Human mesenchymal stem cells (MSCs) are multipotent stromal cells that are capable of self-renewal and differentiation into various lineages mainly including osteoblasts, chondrocytes, and adipocytes. Their major source *in vivo* is the bone marrow, and they have also been found in many other adult tissues such as the adipose tissue, dental pulp, and umbilical cord [1, 2]. In recent years, MSCs have drawn a lot of biomedical research interest for their great potential in regenerative medicine due to their high proliferative ability and lineage plasticity [3]. Many studies have highlighted the promise of somatic MSCs as putative therapeutics for a number of disorders such as osteoarthritis, osteogenesis imperfecta, and even type II diabetes [1, 4, 5]. Compared with embryonic stem cells, since MSCs can be directly obtained from adult individuals, the ethical controversies associated with stem cell therapies are largely eliminated. For a comprehensive review of MSC-based clinical trials conducted worldwide, one may refer to the recent survey [4]. As a side note, in literature, the term MSCs may also refer to mesenchymal stromal cells instead of mesenchymal stem cells [3, 6]. The main reason for this debate on MSC definitions is that the isolation of MSCs according to the current ISCT criteria has produced heterogeneous, nonclonal cultures of stromal cells, including stem cells with different multipotent properties [3, 4]. Since the technical discrimination of mesenchymal stem cells and stromal cells is out of the scope of this paper, we simply use the term MSC to specifically describe a cell with documented self-renewal and differentiation characteristics [3], which is also consistent with the terminology used in the two experimental studies from which we obtain the long-term MSC proliferation data [7, 8].

However, although MSCs may be isolated from a variety of tissue sources, their concentration is still very low. Consequently, it is impossible to collect the large number of MSCs required for clinical trials purely from a single donor, which is one of the major limitations in the medical use of MSCs [9, 10]. Therefore, ex vivo expansion is a necessary step for the acquisition of sufficient MSCs [2, 4], and many research efforts have been devoted to the optimization of MSCs culture protocols, including the composition of the basal culture medium, the addition of specific growth factors, the seeding density, and the biophysical environment [11]. In this study, we focus on the effects of oxygen tension on MSC expansion, which has been investigated by plentiful experimental studies [2, 7, 8, 12–15]. While the typical *in vivo* niche of MSCs, the bone marrow, is characterized by a low oxygen concentration (1% to 7%, *hypoxia*), currently, MSCs are often expanded under the atmospheric oxygen concentration around 21% (*normoxia*) [13]. However, lots of studies have reached a general agreement that hypoxia can extend MSC lifespan and thereby enhance their proliferative efficiency greatly in long-term culture [8, 14, 16–19]. The intracellular molecular mechanism of such effects is still unclear, which may involve oxidative stress [18], gene instability [16], and the regulation of p53 and p16 [18, 20, 21], but many experimental studies imply that in principle, hypoxia promotes MSC long-term expansion by slowing down *replicative senescence*, that is, the inherent division limitation of cultured cells even in an ideal environment [11, 12, 16, 22]. Here, we should remark that, in literature, a sharp definition of senescent cells is still lacking since deep understanding of mechanisms that induce cellular senescence is still missing. In particular, regarding the biological features in senescence of MSCs, Capasso et al. have investigated MSC senescence induced by oxidative stress, doxorubicin treatment, X-ray irradiation, and replicative exhaustion, to determine a specific signature for acute and replicative senescent MSCs with changes in autophagy, proteasome activity, and metabolism [6]. In this study, by cellular senescence, we generally refer to replicative senescence, that is, a limitation in the number of times that normal cells can divide, which is induced by prolonged periods of cellular stress, such as continuous proliferation [23]. Despite the experimental observations about replicative senescence, we may also suspect that such promotion is caused by a higher death rate, a lower division rate, or a faster senescence pace under normoxic conditions. Therefore, to verify this hypothesis and to interpret the experimental findings about hypoxic influence on MSC expansion from a theoretical perspective, we seek to assess the interplay between replicative senescence and hypoxia using mathematical modeling and quantitative analysis.

Mathematical studies of stem cell systems and their population dynamics, such as the computational modeling of Nanog dynamics in mouse embryonic stem cells [24], the stress distribution throughout engineered heart muscles [25], the lineage specification of hematopoietic stem cells [26], and tumor growth [27], have succeeded in providing valuable insights and quantitative description of the underlying biological processes. However, regarding MSC expansion, despite the aforementioned numerous experimental studies, as far as we know, there are only two quantitative modeling studies in literature closely related with the impact of oxygen tension on MSC expansion. Lemon et al. proposed an ordinary differential equation- (ODE-) based model to describe the proliferation and differentiation of human MSCs grown inside artificial porous scaffolds under different oxygen concentrations [28]. However, their study was conducted in the context of 3D culture of MSCs inside scaffolds, and their model was formulated around the limited porous volume and the oxygen-dependent secretion of extracellular matrix (ECM). Obviously, such context differs significantly from the common 2D expansion in labs since cells are usually subcultured before confluence, making space not a limitation. In the other study performed by Krinner et al. [29], an individual cell-based stochastic model was constructed for pellet cultures of MSCs to describe their expansion and chondrogenic differentiation, assuming that the oxygen-dependent cell state fluctuations are reversible. Their model is mainly concerned with MSC lineage commitment and the consequent cell population structure. In short, neither of the two studies focuses on the relation between oxygen tension and replicative senescence explicitly. Hence, a concise and easy-to-understand mathematical model is of great need to help elucidate the underlying mechanism of oxygen influence on MSC expansion *in vitro*.

To this end, the purpose of our study is to develop a powerful, yet highly interpretable mathematical model to describe the long-term population dynamics of MSCs commonly cultured in 2D environment, like Petri dishes, and to evaluate the potential influence of hypoxia on replicative senescence quantitatively. As we shall see, starting from some simple assumptions rooted in experimental observations, our model is sufficient to accurately reproduce the MSC proliferation behavior in long-term culture and to clearly explain the remarkable difference of MSC expansion capacity under hypoxic and normoxic conditions.

## 2. Methods

Ordinary differential equation (ODE) is the most orthodox method to model system dynamics, for example, the classic model for tumor growth predication [27], the computational modeling of megakaryocytic differentiation [30], and mathematical models to study stem cell population dynamics and stem cell niche regulation [31]. In this section, we will first determine the variables in the ODE system depicting MSC expansion according to existing experimental evidence, then build a two-compartment model composed of two cellular states, and conduct subsequent parameter identification, whose details are described in the following subsections.

### 2.1. Model Assumption and Experimental Evidence

Motivated by the fact that MSCs have only a limited lifespan in long-term culture, the main assumption of our model is that oxygen tension can influence the progression of replicative senescence during MSC aging. For example, it was observed that most of the MSCs cultured under normoxic conditions were in senescence after 100 days, while fewer senescent cells were identified for those in hypoxic culture, leading to an additional 8–20 population doublings under hypoxia. Such possible inhibiting effect of hypoxia on replicative senescence has been reported in many experimental investigations, though various reasons were speculated to explain this phenomenon [12, 16]. For instance, Estrada and his colleagues attributed this extended lifespan of MSCs at lower oxygen tensions to the reduced oxidative stress and thereby lessened DNA damage [16]. Other researchers concluded that the change of MSC self-renewal competence was caused by the downregulation of p16 and p21 under hypoxia or the upregulation of p53 under normoxia, supported by observations in both short-term and long-term cultures [17, 18, 21]. In this present work, unlike common experimental studies which try to identify the specific molecules dominating cellular response to hypoxia, we placed our research at the cell population level and designed a mathematical model by taking mainly three factors which may possibly affect cell expansion efficiency into consideration, including the cell division rate, the cell death (apoptosis) rate, and the replicative senescence rate. Then, we attempted to substantiate the statement that the growth advantage of MSCs in long-term expansion under hypoxia is mainly attributed to the delayed replicative senescence by quantitative analysis of experimental data using our mathematical model.

### 2.2. Two-Compartment ODE-Based Model

**Figure 1:**
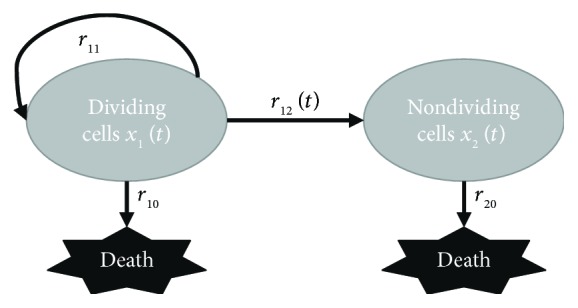
Schematic representation of the two-compartment model. This model conceptualizes the transition of MSCs from the originally dividing state *x*_1_ to the ultimately nondividing state *x*_2_. Cells in both states may die due to internal or external stimuli. *r*_11_ is the division rate, while *r*_10_ and *r*_20_ are the death rate of cells in the two states, respectively, all treated as constants. *r*_12_(*t*) is the time-varying state transition rate.

By its formal definition, a multicompartment model is a mathematical model used to depict the material or energy transmission among the compartments of a system, where each compartment is considered as a homogeneous entity [32]. Based on the experimental observations, there are at least two kinds of MSCs, that is, two homogeneous entities, to be considered in this MSC expansion model: the proliferating and senescent ones. Thus, we developed a two-compartment ODE-based model to incorporate the two cell states and to depict the possible transitions between them. Typically, only few MSCs may differentiate spontaneously in ex vivo expansion unless induced with lineage-specific mediums on purpose [33, 34]. Therefore, cells that cease growth during expansion are mainly senescent ones rather than committed ones. Nonetheless, for comprehensiveness, our model defines two more general cell states, termed *dividing* and *nondividing* cells, of which the latter is mainly composed of senescent cells. Additionally, because replicative senescence is an irreversible permanent cell cycle arrest [22], only cellular state transition from the dividing compartment to the nondividing compartment is allowed in this model. Besides, cells may undergo apoptosis to death in both states. Overall, the concept schematic of our model is illustrated in Figure 1, and the associated governing equations are as follows:

(1) $$\begin{array}{c} {\dot{x}}_{1}\left(t\right)=r_{11}x_{1}\left(t\right)-r_{10}x_{1}\left(t\right)-r_{12}\left(t\right)x_{1}\left(t\right),\\ {\dot{x}}_{2}\left(t\right)=r_{12}\left(t\right)x_{1}\left(t\right)-r_{20}x_{2}\left(t\right), \end{array}$$

where *x*_1_(*t*) and *x*_2_(*t*) represent the dividing and nondividing subpopulations, respectively, whose time derivatives are denoted by ${\dot{x}}_{1}\left(t\right)$ and ${\dot{x}}_{2}\left(t\right)$ correspondingly. In addition, the division rate *r*_11_ of dividing cells and the death rate of the two subpopulations, *r*_10_ and *r*_20_, are all assumed to be time independent, that is, constants. This is a widely adopted hypothesis in cell-modeling studies [30, 35–37], since these parameters usually do not change significantly with time, whereas using constant parameters can greatly simplify the mathematics and hence highlight the most important part of the model.

**Figure 2:**
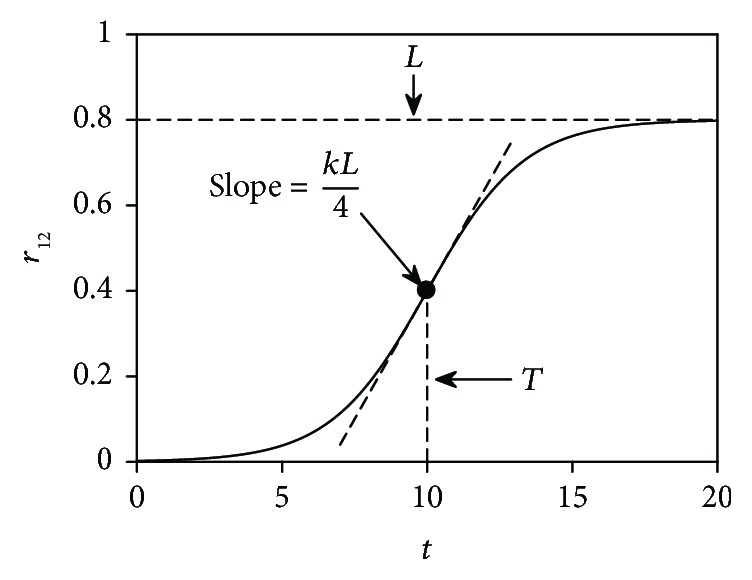
Illustration of the logistic function. It is the form of the state transition rate *r*_12_(*t*) in the two-compartment model, where *T* is the midpoint and *L* specifies the upper bound, while *k* can be used to tune the steepness of the sigmoid curve. The slope of the tangent, that is, the derivative of *r*_12_(*t*) at *t* = *T*, is also shown by the dashed line.

The most interesting and crucial component of our model is the state transition rate *r*_12_(*t*), which is a time-varying function to embody our key assumption about the influence of oxygen tension on replicative senescence. It should be emphasized that, at the population level, senescence is a continuous process instead of a simple off/on binary switch due to the heterogeneity of cells [37–39]. Furthermore, taking the essential features of senescence into account [22, 38], we require the function *r*_12_(*t*) to possess another two properties: (i) it should be low at the early stage and increases monotonically as cells age and (ii) it must have a limited upper bound for biological feasibility. Thus, the well-known logistic function came to our mind as a qualified candidate for state transition rate *r*_12_(*t*), whose general form is as follows:

(2) $$\begin{array}{c} r_{12}\left(t\right)=\frac{L}{1+e^{-k\left(t-T\right)}}, \end{array}$$

where *L* denotes the upper bound, *k* depicts the steepness, and *T* is the midpoint of the sigmoid curve. Visually, *r*_12_(*t*) is shaped by these three parameters, as depicted in Figure 2 using fictitious parameter values as an example.

In experiments of MSC expansion *in vitro*, usually only the total number of cells can be counted directly instead of the two separate subpopulations. Let *y*(*t*) be the total cell number, which is the sum of *x*_1_(*t*) and *x*_2_(*t*). To facilitate the subsequent parameter identification, the two constants, *r*_11_ and *r*_10_, in (1) are combined into a single quantity, *r*_1_≜*r*_11_ − *r*_10_, denoting the *net division rate*. Then, the overall MSC proliferation model in (1) can be rewritten into a concise state-space form by the following:

(3) $$\begin{array}{c} \left[\begin{array}{c} {\dot{x}}_{1}\left(t\right)\\ {\dot{x}}_{2}\left(t\right) \end{array}\right]=\left[\begin{array}{cc} r_{1}-r_{12}\left(t\right) & 0\\ r_{12}\left(t\right) & -r_{20} \end{array}\right]\left[\begin{array}{c} x_{1}\left(t\right)\\ x_{2}\left(t\right) \end{array}\right],\\ y\left(t\right)=x_{1}\left(t\right)+x_{2}\left(t\right), \end{array}$$

which is essentially a time-variant second-order linear system due to the time-varying nature of *r*_12_(*t*). Generally, we cannot find a closed-loop solution for *y*(*t*) in such systems. Instead, it can be solved by numerical methods [40].

### 2.3. Model Parameter Fitting

There are five parameters to be determined in the model (3). We thereby gather them into a parameter vector, $\theta =\left[r_{1},\;r_{20},\;\begin{array}{cc} \begin{array}{cc} L, & k \end{array}, & T \end{array}\right]$, and then identify their values by fitting to the experimental data *D* = {*y* ^*m*^(*t*_*i*_),  *i* = 1, 2,…, *N*}, where *y* ^*m*^(*t*_*i*_) is the total cell number measured at *t*_*i*_. The parameters are estimated in the common least-squares sense, that is, to minimize the following cost function:

(4) $$\begin{array}{c} J\left(\theta \right)=\overset{N}{\underset{i=1}{\sum }}{\left(y\left(t_{i}\right)-y^{m}\left(t_{i}\right)\right)}^{2}, \end{array}$$

which is the sum of squared residuals. Moreover, we must enforce the biological feasibility of these parameters by imposing proper constraints. Obviously, the three rate parameters *r*_1_, *r*_20_, and *L* should all be positive but cannot be too large for a practical biological system. Besides, a too large steepness *k* will make the logistic function *r*_12_(*t*) behave more like a step function (Figure 2), which is undesirable because as aforementioned, senescence is a continuous process instead of an abrupt change.

To summarize, after imposing constraints on the parameters, the model parameter fitting is formulated as a constrained optimization problem denoted by the following:

(5) $$\begin{array}{c} \min\;J\left(\theta \right)=\overset{N}{\underset{i=1}{\sum }}{\left(y\left(t_{i}\right)-y^{m}\left(t_{i}\right)\right)}^{2},\\ \text{Subject to}\;\left\{\begin{array}{l} 0<r_{1}<1,\\ 0<r_{20}<1,\\ r_{1}<L<1,\\ 0<k<1,\\ 0<T<t_{s}, \end{array}\right. \end{array}$$

where *r*_1_, *r*_20_, *L*, and *k* are all assigned a very loose upper bound and *t*_*s*_ is the time when most cells are observed to cease proliferation experimentally. The additional relation *r*_1_ < *L* stems from the stability requirement of the system (3), that is, we require *r*_1_ − *r*_12_(*t*) < 0 for certain *t* > *t*_*c*_ such that the cell population is prohibited from explosion to infinity [41].

**Figure 3:**
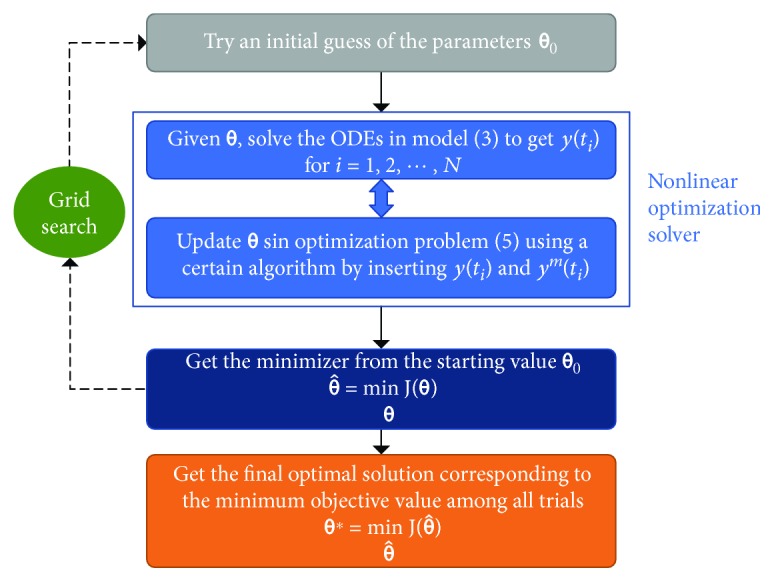
Workflow of parameter fitting via nonlinear regression. First, given initial conditions, we can solve the time-variant ODEs in our model (3) by numerical ODE solvers to get the model predications *y*(*t*_*i*_) for *N* time points in interest. Then, the constrained optimization problem (5) for parameter fitting can be tackled with iterative nonlinear optimization algorithms, for instance, the Nelder-Mead simplex search approach (like the *fminsearch*, *fmincon*, or *lsqcurvefit* functions in MATLAB). Here, it should be noted that to avoid the possible bad local minima associated with nonlinear, nonconvex optimization problems, we may need to try multiple initial guesses of the parameter vector **θ**_0_. We use a systematic approach based on grid search to coordinate multiple initial value trials [44], thanks to the low dimension and small dataset size in this study. This approach can increase the probability that we find the global minimum or at least a good local minimum close to the global one. A detailed description of the fitting procedures is provided in Supplementary Materials (available here).

By definition, the optimization in (5) is a nonlinear regression problem, which is generally solved through successive iterations [42]. First, given initial conditions, the numerical solution of *y*(*t*) in the model (3) for each time point *t*_*i*_ can be obtained by numerical ODE solvers like the *Runge-Kutta* method. Then, after we get *y*(*t*_*i*_), we can tackle the optimization problem (5) with some iterative nonlinear optimization algorithms such as the *Nelder-Mead* simplex search approach. The general workflow of parameter fitting through nonlinear optimization in this study is shown in Figure 3. For practical implementations, we may resort to MATLAB (The MathWorks Inc.) and use its built-in functions such as *ode45*, *fminsearch*, and *fmincon* [27, 28, 43]. More details will be covered in the following parts with respect to specific datasets.

## 3. Results

In this section, we first investigated our model with two experimental datasets collected from MSC long-term proliferation *in vitro* to examine whether this model could reproduce the observed growth curves and explain the disparity of growth capacity under hypoxia and normoxia. After that, a sensitivity analysis study was performed to reveal which of these parameters have the most significant influence on the population dynamics.

### 3.1. Collection of Experimental Data

We considered two experiments in literate regarding long-term expansion and differentiation of human MSCs, termed *experiment A* and *experiment B* hereafter. In experiment A, Fehrer and her colleagues cultured MSCs up to about 120 days at 3% oxygen (hypoxia) and 20% oxygen (normoxia), respectively [7]. In experiment B, to develop a superior protocol for MSC expansion by combining low-density and hypoxic culture, Tsai et al. studied MSCs from 3 donors under normoxic (21% oxygen) and hypoxic (1% oxygen) conditions for about 90 days [8]. Since we focus on MSC expansion in this study, only the proliferation data from the two studies without differentiation induction were adopted. To automatically and accurately extract the experimental data published as figures in these two articles [7, 8], the online tool *WebPlotDigitizer* v3.12 (https://automeris.io/WebPlotDigitizer/) was utilized.

### 3.2. Experiment A

**Figure 4:**
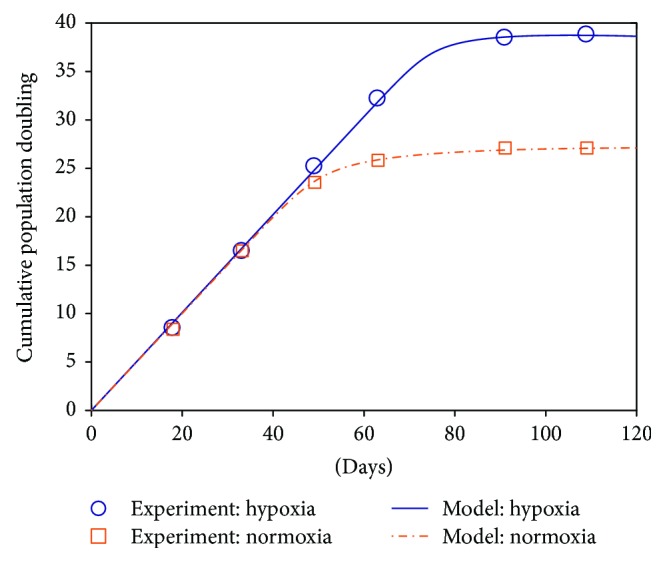
Experimental measurements and model-fitted population dynamics of MSCs under hypoxia and normoxia in experiment A. The cultured MSCs under study were originally obtained from a female donor of age 56. PD: cumulative population doublings.

The experimental data was extracted from Figure 3(a) in the paper [7] including two growth curves of bone marrow-derived MSCs, one from a female donor of age 56 and the other from a 78-year-old male donor, expanded in both oxygen conditions. Since the two datasets are quite similar, here we only show the model fitting and analysis results for the first growth curve (from the female donor of age 56), which includes six measurements for each oxygen condition (Figure 4), and put the other in the Supplementary Materials (Figures S1–S3, Table S1). In consistency with the original paper [7], in this study, the cell number is evaluated by cumulative population doublings (PD), an analogy to the logarithmic scale, defined as follows:

(6) $$\begin{array}{c} y_{PD}\left(t\right)=\log_{2}\frac{y\left(t\right)}{y\left(0\right)}, \end{array}$$

where *y*(0) is the initial cell number, *y*(*t*) the cell number, and *y*_PD_(*t*) the corresponding PD at time *t*.

Now with the six measurements *y*_PD_^*m*^(*t*_*i*_) at hand, *i* = 1, 2,…, 6, to conduct model parameter fitting, we additionally need the initial cell numbers of the two subpopulations, *x*_1_(0) and *x*_2_(0), to solve the ODE model (3). However, typically in practice, the precise value of *x*_1_(0) and *x*_2_(0) cannot be measured directly. Fortunately, at the beginning, senescent cells only took up a very small portion, observed by checking their morphological appearance, which was further confirmed through assessment of specific senescence markers [7]. Therefore, we simply assumed that all cells were dividing initially, that is, *x*_1_(0) = *y*(0) and *x*_2_(0) = 0. Then, we can fit the five parameters in our model (3) by minimizing the constrained cost function in (5) with nonlinear least-squares optimization algorithm such as *fmincon* or *fminsearch* in MATLAB. The fitted parameters for the two conditions are listed in Table 1. By inserting these fitted parameter values, we simulated the MSC proliferation process numerically using the model (3). Figure 4 shows the comparison between model-predicted and experimentally measured MSC expansion dynamics under hypoxia and normoxia.

Notably in Figure 4, our model can fit the experimental data in both two conditions nearly perfectly. This impressive fitting performance implies that the logistic function is a good candidate to model the oxygen tension-dependent and time-variant replicative senescence progression process. With this key component, our two-compartment model can well describe the MSC proliferation dynamics in both oxygen conditions with enough explanatory power. Although other functions like the generalized logistic model and the Gompertz model can also display a sigmoid curve, we choose the simple logistic function in our model according to the Occam's razor principle. It is also noted that more complex functions usually have more parameters, which may compromise the interpretability of the theoretical model since it is hard to endow all the parameters with proper biological meanings.

**Figure 5 fig5:**
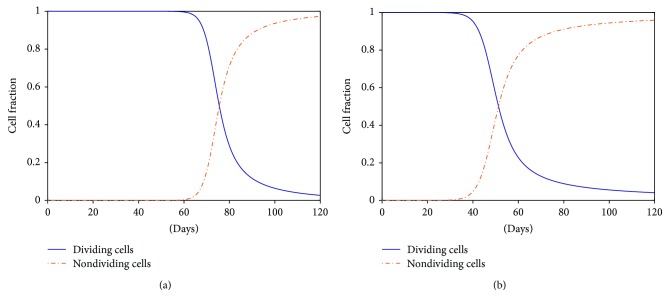
Simulation of the dividing and nondividing cell fractions in the two oxygen environments of experiment A. (a) Hypoxia. (b) Normoxia. Initially (at day 0), it is assumed that all cells are dividing in both two conditions.

To further verify this argument, we examined the proportion of the dividing and nondividing cells in the whole culture process with data simulated by our model (3), presented in Figure 5. Apparently, almost all MSCs are dividing at the early stage, while the nondividing fraction keeps increasing until most of the cells cease division at the end. Therefore, in agreement with the qualitative experimental findings [7], at population level cells gradually lose their proliferative capability along with their aging. Furthermore, Figure 5 shows that cells under normoxia cease growth much earlier than hypoxic cells. After approximately 50 days, only half of the cells keep dividing under normoxia (Figure 5(b)), while such decline does not happen until about 75 days in hypoxic culture (Figure 5(a)). There is no doubt that such delay of replicative senescence about 25 days will contribute to significantly more population doublings in MSC expansion. This fact further confirms that replicative senescence is indeed slowed down under hypoxia in consistency with multiple experimental observations that considerably more senescent cells are detected in normoxic culture [7, 8, 20, 33].

**Figure 6:**
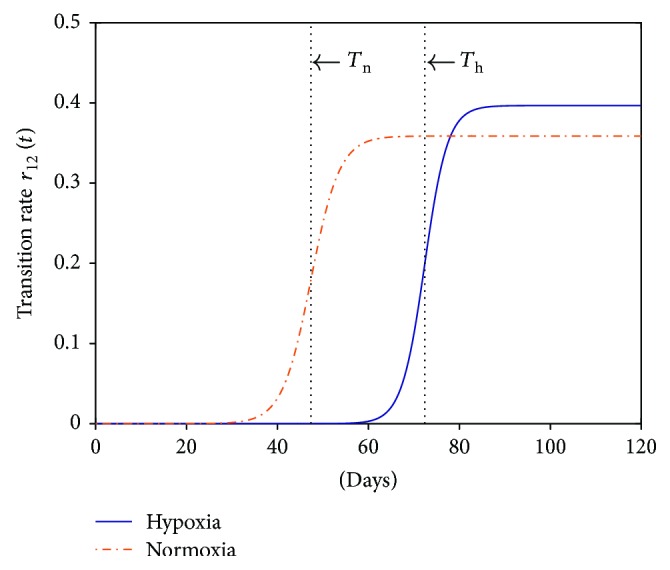
Comparison of the time-variant state transition rate *r*_12_(*t*) for MSCs cultured under hypoxia and normoxia in experiment A. The three parameters used to simulate the logistic function *r*_12_(*t*) defined in (2) can be found in Table 1, of which the midpoint time *T* is annotated in the figure as *T*_n_ and *T*_h_ for normoxia and hypoxia, respectively.

To acquire a deeper insight of the above observed phenomena, we continued to probe the time evolution of the state transition rate *r*_12_(*t*), defined in (2), under the two oxygen conditions.  Figure 6 shows the comparison of the state transition rate *r*_12_(*t*) simulated numerically using the two sets of parameters in Table 1 for hypoxic and normoxic conditions, respectively. To assess the pace of replicative senescence quantitatively, the midpoint time *T* may act as a good indicator of the senescence pace or roughly the *onset* of massive senescence at population level, since replicative senescence is a continuous process. We notice *T*_n_ = 47.35 and *T*_h_ = 72.37 for normoxia and hypoxia, respectively, which clearly exposes the delay of replicative senescence under hypoxia. Here, though the steepness *k* is close, one may be still wondering the role of *L* since it looks somewhat different in the two conditions as well (Figure 6). To further check its effect, we deliberately set *T*_h_ = *T*_n_ in the two conditions and ran simulations again. Results show that, unlike Figure 4, the model predications under hypoxia with such parameters deviate far away from the experimental data (Figure S4). Thus, this inconformity proves the dominant role of *T* in causing MSC expansion disparity in the two conditions. In fact, it is easy to see from Figure 2 that a larger *L* value can only tend to speed up senescence and thereby inhibit MSC expansion instead of promotion.

As a concluding remark of experiment A, our model tells that the distinct MSC proliferation efficiency mainly results from the different senescence pace, indicated by the large difference of *T*, in the two conditions. Besides, although we simply assume all cells are dividing initially in the present results, the fitted parameter values will remain almost unchanged and therefore our reasoning still applies, even if the initial fraction of nondividing cells is not zero, say, 10%, as shown in Table S2. Next, we will further highlight the competence of our model with another dataset collected from long-term MSC expansion, whose data is not as complete as this one.

### 3.3. Experiment B

**Figure 7:**
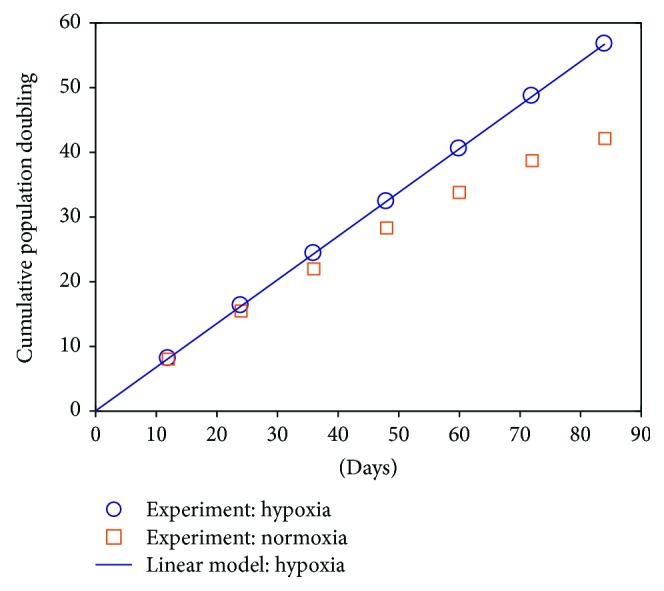
Experimental measurements of cell numbers under hypoxia and normoxia and the fitting of the hypoxic data with a linear model in experiment B. The *R* ^2^ (coefficient of determination) of this simple linear regression is very close to 1, indicating that the hypoxic data can be well replicated with the linear model.

Generally, only after a long time of expansion, roughly more than 100 days or 15 passages [7, 16], will MSCs expanded *in vitro* completely stop proliferation. Consequently, quite few experimental data are available in literature for such a long time. The data of experiment B were extracted from Figure 1(a) in the paper [8] with a culture period of only 84 days. For consistency, the cell number is also measured using PD instead of the original fold increase [8], which can be obtained with the following formula:

(7) $$\begin{array}{c} y_{PD}\left(t_{k}\right)=\log_{2}\frac{y\left(0\right)\Pi_{i=1}^{k}f\left(t_{i}\right)}{y\left(0\right)}=\overset{k}{\underset{i=1}{\sum }}\log_{2}f\left(t_{i}\right),\;k=1,2,\ldots ,7, \end{array}$$

where *f*(*t*_*k*_) is the fold increase of cell number at time *t*_*k*_. After such data preprocessing, the experimental data of experiment B including seven measurements are shown in Figure 7. We notice that in neither of the two conditions have the expanded MSCs reached the stationary growth phase, that is, they are still proliferating even at the end, which will inevitably cause difficulty in model parameter fitting, especially for the hypoxic case. To examine the universality of the model structure and model characteristics we found in experiment A, we tried to combine the hypoxic and normoxic data together to identify the model parameters.

Now recall our findings in experiment A (Figure 4). If most of the cells are actively dividing, particularly under hypoxia, the cell population will exhibit an exponential growth law, which appears as a straight line when cell numbers are represented by PD, justified by the following:

(8) $$\begin{array}{c} y_{PD}\left(t\right)=\log_{2}\frac{y\left(t\right)}{y\left(0\right)}\approx \log_{2}\frac{y\left(0\right)e^{r_{1}t}}{y\left(0\right)}=\left(r_{1}log_{2}e\right)t, \end{array}$$

where *y*(0) is the initial cell number and *r*_1_ is the net expansion rate of dividing cells. In (8), we simply assume that all cells are dividing such that there exists $\dot{y}\left(t\right)={\dot{x}}_{1}\left(t\right)=r_{1}$. Unsurprisingly, we notice that the hypoxic data points in Figure 7 indeed seem to lie on a straight line. Therefore, it is natural to perform linear regression on the hypoxic data to fit the straight line's slope *s* = *r*_1_log_2_ *e* (Figure 7).

In experiment A, the parameter *r*_1_ we have fitted in the two conditions are approximately equal (Table 1). Thus, it is reasonable to assign *r*_1_ = *s*/log_2_ *e* obtained under hypoxia to its normoxic counterpart. With *r*_1_ fixed, it is feasible to fit the remaining four parameters with the normoxic data. Nevertheless, for hypoxia, because all experimental data belong to the exponential growth phase, it is still impossible to determine a *unique* optimal set of parameters by fitting the hypoxic data. To resolve this difficulty, we chose *r*_20_, *L* under hypoxia identical to the ones we have fitted for normoxia based on our experience in experiment A (Table 1) and tuned the parameter *k* manually to match the exponential grow curve. The parameter fitting results for experiment B are reported in Table 2.

**Figure 8:**
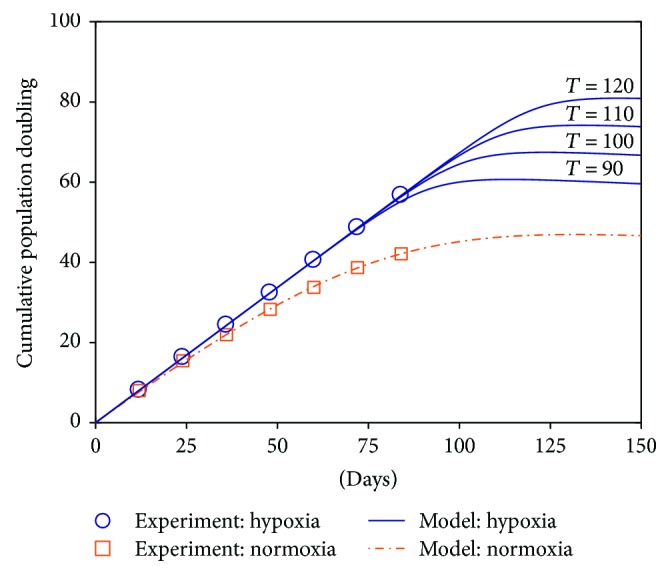
Experimental measurements and model-predicted population dynamics of MSCs under hypoxia and normoxia in experiment B. For the series of growth curves under hypoxia, the first four parameters are fixed (see Table 2), while the last parameter *T* varies from 90 to 120 equidistantly to demonstrate its significant effect on cell proliferation. PD: cumulative population doublings.

With the parameter values listed in Table 2, we can simulate our model to get the proliferation trajectory for MSCs under normoxia, shown in Figure 8. Once again, the theoretical predications generated by our model, even fitted with incomplete data (i.e., cells are still proliferating at the end), show good agreement with the experimental measurements. Since the parameter *T* remains unknown for hypoxia (Table 2) due to the fact that all MSCs under hypoxia are still in the exponential growth phase, we tested different values of *T* to highlight its impact on population dynamics (Figure 8). Apparently, models with *T* = 100, 110, or 120 can match the experimental data equally well. More interestingly, this cluster of growth curves, varied by only one parameter *T*, exactly demonstrates the paramount influence of *T* on MSC proliferative capacity: a delay of *T* by 10 days can bring about roughly an additional 7 PD under hypoxia. This is consistent with our key findings in experiment A, that is, the distinguished MSC expansion ability in long-term culture under hypoxia and normoxia is mainly reflected by the different values of parameter *T* in our model under these two conditions.

To summarize our work in experiment B, we have first successfully fitted our model with only experimental data before the stationary stage. It is a critical capability of our model to accomplish not only interpolations but also extrapolations with even poor availability of experimental data. This fact reflects that our model has grasped the dominating cellular mechanism responsible for the experimental observations. On the other hand, we must admit that it is unattainable to determine a unique set of model parameters if too little diversity is present in the data because many sets of parameters can work equally well. For example, with only exponential-phase cell proliferation data under hypoxia in this experiment, multiple possible values of *T* lead to the same fitting performance (Figure 8). Even so, by examining various parameter values tentatively, our model still reveals the pivotal impact of oxygen tension on MSCs expanded *in vitro*: hypoxia can delay the onset of MSC senescence and thus promote their proliferation, implied by the great influence of *T*. Finally, as a side note, one may notice that the parameter values for Experiments A and B are different. This discrepancy stems from many other variables between the two experiments (from two independent studies [7, 8]), for example, the specific culture condition, the growth media, and the age/sex of the cell donors. It is reasonable to obtain different parameter values since all these variables may influence MSC expansion efficiency. However, in each experiment (A or B), the only control variable between the two conditions (hypoxia and normoxia) is the oxygen tension, and after fitting our model to the data, the value of the parameter *T* under hypoxia is consistently much larger than the one under normoxia for both experiments.

### 3.4. Sensitivity Analysis

In above experiments, our two-compartment model can effectively describe MSC expansion by approximating replicative senescence with a logistic transition function. To further assess the impact of parameters on model outputs, that is, to evaluate which parameters are the most influential on the system output when the inputs (the initial condition) are fixed, we investigated their individual sensitivity using one of the most widely used evaluation methods, called *one-at-a-time* [45]. In this strategy, only one parameter is varied at a time while all others are fixed at their nominal (fitted) values; then, the corresponding change of model output is recorded [37, 45]. To quantify the parameter sensitivity, each of the five parameters is perturbed in turn by a random degree *ϵ*, where *ϵ* is uniformly distributed between −10% and 10%, and the other parameters are fixed at their nominal (fitted) values. Repeat the parameter perturbation and subsequent model simulation for *K* = 1000 times. Then, the sensitivity *s*_*i*_ of the *i*th parameter is scored by the standard deviation of the model output *y*_*sp*_ (population doublings at the stationary phase) in the total *K* runs, given as follows:

(9) $$\begin{array}{c} s_{i}=\sqrt{\frac{1}{K-1}\overset{K}{\underset{j=1}{\sum }}{\left(y_{sp,j}-\mu \right)}^{2},} \end{array}$$

where *y*_*sp*,*j*_ is the model output of the *j*th run and *μ* = 1/*K*∑_*j*=1_^*K*^ *y*_*sp*,*j*_ is the mean of *y*_*sp*_ in the *K* runs.

**Figure 9:**
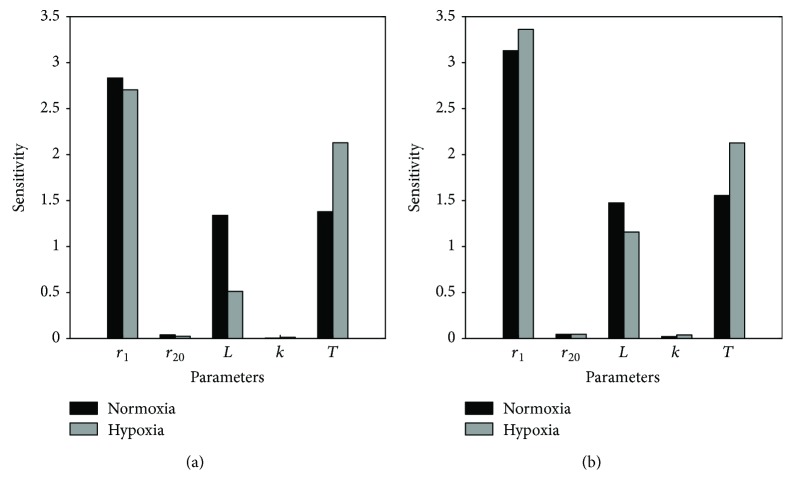
Parameter sensitivity analysis of the two-compartment model. To evaluate the sensitivity, the parameters are perturbed around their nominal values fitted in experiment A with cell data collected from two donors in two conditions: (a) MSCs obtained from a female donor of age 56 and (b) MSCs isolated from a 78-year-old male donor. The exhibited sensitivity is measured by the standard deviation of model outputs in 1000 simulations with randomly perturbed (between ±10%) parameter values using the *one-at-a-time* approach.

In experiment A, we have enough experimental data and have fitted all the parameters for datasets of two donors, that is, one female donor of age 56 (Table 1) and another male donor of age 78 (Table S1). Thus, the sensitivity analysis was conducted using the model obtained in experiment A. The outcome is summarized in Figure 9. Despite the various parameter values in the four cases, their sensitivity distribution shares a notably common pattern, demonstrating the inherent consistency of the parameter sensitivity ranking of our model, even though the absolute values of these parameters are different. This fact indicates that the interpretation of our model is reasonable even in different experimental settings. Regarding the individual sensitivity, as expected, the fundamental driving force of MSC proliferation is the net expansion rate of dividing cells, *r*_1_, highlighted as the most influential parameter, which dominates the exponential growth phase, as demonstrated in (8). Apart from *r*_1_, the second predominant parameter is *T*, which largely determines the MSC state transition rate from the dividing state to the nondividing state *r*_12_(*t*). On the contrary, the death rate *r*_20_ and the steepness parameter *k* only exhibit negligible sensitivity (Figure 9).

In brief, though the two parameters *r*_1_ and *L* also possess noteworthy influence on model outputs (Figure 9), it is only the parameter *T* that can explain the impressive difference of the model output, that is, cell number, under hypoxic and normoxic conditions (Figure 4, Figure S1) since *r*_1_ and *L* have close and comparable values in the two conditions (Table 1, Table S1). However, one may also notice that the value of *r*_1_ is slightly higher under the hypoxic condition (Table 1) and may suspect its role in promotion of MSC expansion. To clarify this point, we made another simulation study by exchanging the *r*_1_ values of the two conditions while leaving the other parameters unchanged. The result is shown in Figure S5. By comparing Figure S5 with Figure 4 in the main text, we can see that the fitting results change quite little, while there is still a large gap between the final population size under the two conditions. Thus, we can conclude that the minute difference of the fitted *r*_1_ values plays just a negligible role and cannot explain the significant difference of MSC expansion under the two oxygen conditions. Essentially, the largest sensitivity of *r*_1_ is attributed to the exponential growth law in the early stage, see (8). However, the *r*_1_ values under the two conditions are so close that they cannot explain the considerable difference in MSC proliferation. As for the parameter *L* which also has a large sensitivity, it is easy to see from Table 1 and Figure 6 that the larger *L* value under hypoxia can only speed up the cellular senescence process and thereby impede MSC expansion. Thus, this parameter is definitely not the factor that contributes to MSC proliferation boosting. By eliminating these possibilities, we can be certain that it is only the parameter *T* (increased around 50% from normoxia to hypoxia) that can explain the impressive difference of the cell number under hypoxic and normoxic conditions.

In summary, with the above analysis of the two parameter sets fitted by MSC expansion data under hypoxia and normoxia, respectively (Table 1, Table 2, and Table S1), we can conclude that it is the parameter *T*, a qualified indicator of replicative senescence pace, that is responsible for the distinct MSC expansion productivity under different oxygen conditions. Thus, combining the parameter magnitude comparison and the sensitivity analysis has further strengthened our main argument and verified the fundamental hypothesis of our model, that is, hypoxia can promote MSC expansion through slowdown of replicative senescence, from another perspective, indicated by the impressive sensitivity of *T* as well as the considerable difference of its value under two oxygen conditions.

## 4. Discussion

Mesenchymal stem cells are considered valuable and easily accessible cell sources for regenerative medicine due to their high proliferation capacity, their potential for multiple differentiation pathways, their active paracrine effects, and their immunomodulatory features for suppression of excessive immunoreactivity [4, 46–48]. Generally, cell-based therapies and tissue engineering require a sufficiently large number of high-quality cells. Accordingly, the efficient ex vivo expansion of MSCs is of critical importance and has drawn a lot of research interest. In literature, the influence of oxygen tension on MSC expansion and differentiation has been investigated extensively by many experimental studies (see review [12, 49]). Despite the abundance of experimental studies, to the best of our knowledge, our work in this study is the first attempt to analyze the effects of hypoxia on MSC expansion in long-term 2D culture through mathematical modeling in a purely data-driven way. Mathematical reasoning and simulation results have demonstrated that hypoxia can postpone replicative senescence and consequently extend the lifespan of MSCs to produce more cells.

There are multiple suspicious factors which might account for the MSC growth disadvantage when subject to a high oxygen tension. For example, we may speculate about a higher death rate or a lower cell division rate under normoxia, considering the typical *in vivo* low-oxygen environment in the bone marrow. To determine the contribution of such factors, we have encoded these hypotheses as parameters in the two-compartment model and then identified parameter values by fitting to the experimental data. Results show that the net expansion rate *r*_1_ and the death rate *r*_20_ have similar values in both oxygen conditions, implying that the negligible changes of cellular net division rate and death are unlikely to be the main cause of MSC expansion variations. This finding is in agreement with experimental observations: though cell viability might be slightly higher under hypoxia, no obvious differences regarding necrosis or apoptosis were detected experimentally [7, 50]. Besides, MSCs expanded *in vitro* can typically maintain a stable undifferentiated phenotype over time without tumorigenesis [33, 48]. Therefore, the plausible variations in the proportion of committed cells arising from different oxygen tensions cannot explain the dramatically reduced MSC expansion under normoxia, either. By contrast, numerous studies have reported that exposure of MSCs to normoxia can contribute decisively to replicative senescence. They observed a clearly greater number of enlarged and flattened cells, the typical morphology of senescent cells, a higher expression level of senescence markers like SA-*β*-gal, as well as a shorter telomere length under normoxia than hypoxia, while such phenomena would not appear until many more days later under hypoxia [7, 8, 20, 21]. In our model, because the upper bound *L* of the state transition rate *r*_12_(*t*) in (2) are similar in the two oxygen conditions (Table 1 and Table 2), it is reasonable to consider the midpoint time parameter *T* as a representative measurement of the replicative senescence pace. Next, the fitting results and sensitivity analysis tell that the parameter *T* varies most between hypoxia and normoxia and possesses very high sensitivity, indicating that the most important variable leading to MSC expansion difference in low and high oxygen tensions resides in the replicative senescence progression. In summary, all the above experimental evidence justifies the findings of our two-compartment model, which state that hypoxia can greatly enhance MSC proliferation and prolong their lifespan mainly through inhibition of replicative senescence in long-term culture.

In retrospect, we notice that MSC proliferation displays the typical *S*-shaped growth curve, which may also be represented by others like the exponential-linear or Gompertz model [27]. However, we must emphasize that such models were originally designed to describe the overall population behavior with no discrimination of various cell states. As a result, though such models with only a single-cell state may also fit the data satisfactorily, they cannot reveal the inherent reason which leads to the experimental observations. For example, the cause of the remarkably different MSC expansion capacity under hypoxia and normoxia would still be obscure in a single-compartment model. Thus, the primary merit of our model does not lie in its good fitting of numerical values, though it has demonstrated excellent fitting performance, but rather in its capacity to interpret the qualitative characteristics, to reveal the biological mechanism and to advance our understanding of the experimental data. Intuitively, we use the logistic function to approximate the replicative senescence progression process during cell aging. Thanks to its high interpretability, the profound impact of hypoxia on replicative senescence has been uncovered and confirmed convincingly.

Technically speaking, the model we have developed in (1) is more of an explanatory model than a predicative one. Explanatory modeling refers to the application of models to data for testing causal hypothesis about theoretical constructs [51]. The top priority of explanatory models is their explanatory power, while predicative modeling focuses on predicative power or *generalization* [51]. Thus, in this study on explanatory modeling, it is the construction and structure of the model based on biological hypothesis that matters most, because the acquired model must be highly interpretable and the parameters should be endowed with biological meanings. This also constitutes our main contribution: we discovered that hypoxia can promote MSC expansion by delaying MSC replicative senescence merely through quantitative analysis of proliferation data with a properly designed two-compartment model, with no need of extra measurements using devoted biological assays, like the detection of cell viability and senescence markers, which may be expensive and time-consuming. Thus, this model is valuable in providing theoretical support for experimentalists' observations. In short, though predictive modeling is forward-looking, explanatory modeling is retrospective [51]. Since our model is not developed for predication purpose, an independent validation experiment is not necessary to test its generalization performance. However, the explanatory power of our model has been validated on multiple experimental datasets, which all lead to the same conclusion: hypoxia causes MSC expansion boosting by inhibiting cellular senescence. With an explanatory model, our study mainly focuses on the comparison of model parameter values fitted under hypoxia and normoxia to explain the underlying mechanism. Thus, it is the relative magnitude of parameters instead of their absolute value that matters.

To enhance MSC expansion to get enough cells for clinical applications, it is of great interest to determine the optimal oxygen concentration so as to maximize the cell yield. However, due to lack of experimental data, the oxygen tension only appears in our model implicitly in the form of hypoxia and normoxia instead of continuous values. Besides, we want to point out that hypoxia and normoxia refer to two coarse ranges of oxygen tension instead of precise concentration values: the former depicts the *in vivo* environment and the latter represents the atmospheric environment. Consequently, we can only demonstrate the superiority of hypoxia against normoxia for MSC long-term expansion, while with the currently available data it is impossible to obtain an accurate predication relationship associating the oxygen tension with the number of MSCs at a given time point. Nevertheless, once we acquire more MSC expansion data systematically at a sequence of oxygen concentrations from dedicated experiments, our model can be extended readily to describe the numerical relationship between oxygen tension and MSC proliferation explicitly. That is, the proposed two-compartment model can serve as a good prototype to be extended into a truly predicative model. Afterwards, the extended model can be used to optimize the oxygen environment and even to build a closed-loop control system for more efficient large-scale cell production by tuning the oxygen tension precisely in real time [52].

Though our model successfully demonstrates that the promoted expansion and the prolonged lifespan of MSCs are mainly attributed to the slowdown of replicative senescence at a reduced oxygen tension, we must admit that it is still a coarse-grained model at the population level and cannot establish the intracellular molecular mechanism for the relevant regulation. In fact, the involved signaling pathways are still controversial and remain to be elucidated, though many speculate that the key response to hypoxia is mediated by the hypoxia-inducible factor- (HIF-) 1 and involves the interaction between p21, p16, and reactive oxygen species (ROS) [8, 12–14, 17, 20]. Thus, a multiscale model integrating the population dynamics and the intracellular biochemical reactions is highly desired to completely dissect the influence of hypoxia on MSC proliferation and differentiation. Further theoretical work on involved signal transduction pathways and gene regulatory networks are planned in our future studies. Lastly, we want to point out that in literature, the effects of hypoxia on short-term MSC proliferation may vary in different studies, which depend on the concrete oxygen tension, the culture conditions, and the cell sources. However, the benefits hypoxia can bring to long-term expansion of MSCs are consistent among various studies even with different culture media and MSC sources [14, 16–19, 49]. As we have addressed since the beginning, our two-compartment model applies specially to the MSC long-term expansion, where the interplay between hypoxia and replicative senescence plays the most important role in determining cell yield.

## 5. Conclusion

This study underlines the influence of oxygen tension on MSC long-term expansion, and we have presented here, for the first time, a comprehensive two-compartment ODE-based model to characterize the MSC population dynamics under hypoxia and normoxia. A unique aspect of the current study is the adoption of the logistic function to depict the time-variant state transition rate between the two compartments, which is a natural analogy to the continuous senescence progression along with cell aging. Though simplistic in nature, our model has captured the key characteristics of the MSC expansion process and provides a computational basis for deep understanding of the oxygen impacts on MSC proliferation. In accordance with experimental evidence, this theoretical model supports the idea that hypoxia can greatly enhance the proliferation of MSCs and prolong their lifespan in long-term culture through inhibition of replicative senescence. Over the course of this study, we have demonstrated mathematical modeling and analysis as a useful tool for quantitatively interpreting and mining the information hidden in raw experimental data. Since our model is designed and fitted with MSC expansion data in 2D culture (Petri dishes), where oxygen is distributed uniformly in the culture media, it deserves efforts to further explore whether this model can be generalized to the 3D culture scenario such as scaffolds.

## Figures and Tables

**Table 1:** Model parameter values for normoxic and hypoxic conditions fitted from MSC expansion data collected in experiment A. *r*_1_: net expansion rate; *r*_20_: death rate of nondividing cells; *L*, *k*, and *T* are the upper bound, the steepness, and the midpoint time of the logistic state transition rate *r*_12_(*t*), respectively.

Parameter	Normoxia	Hypoxia
*r*_1_ (day^−1^)	0.3472	0.3505
*r*_20_ (day^−1^)	0.0133	0.0183
*L* (day^−1^)	0.3588	0.3967
*k*	0.3169	0.3944
*T* (day)	47.3494	72.3664

**Table 2:** Model parameter values for normoxic and hypoxic conditions fitted from data in experiment B. *r*_1_: net expansion rate; *r*_20_: death rate of nondividing cells; *L*, *k*, and *T* are the upper bound, the steepness, and the midpoint time of the logistic state transition rate *r*_12_(*t*), respectively.

Parameter	Normoxia	Hypoxia
*r*_1_ (day^−1^)	0.4679^†^	0.4679^†^
*r*_20_ (day^−1^)	0.0286	0.0286^‡^
*L* (day^−1^)	0.5864	0.5864^‡^
*k*	0.0404	0.15^§^
*T* (day)	79.0046	?^¶^

^†^Fitted from hypoxic data using linear regression and set *r*_1_ under normoxia equal to the one for hypoxia. ^‡^Set equal to their counterparts under normoxia. ^§^Tuned manually to fit the hypoxic data. ^¶^To be determined.

## Data Availability

The data used to support the findings of this study are available from the corresponding author upon request.

## Supplementary Materials

Supplementary Materials The analysis results for the other dataset (with MSCs from a 78-year-old male donor) are presented in Table S1 and Figures S1–S3, corresponding to Table 1 and Figures 4 [Fig fig5]–6 in the main text. Table S2: model parameter values for normoxic and hypoxic conditions fitted from data in experiment A but with 10% nondividing cells at the beginning. Figure S4: model fitting results by setting *T*_h_ = *T*_n_ in experiment A as a comparison to Figure 4 in the main text. Figure S5: model fitting results by exchanging the net expansion rate *r*_1_ in Table 1 as a contrast of Figure 4 in the main text. The detailed fitting procedures are also presented.
