# Supplementary material for: Mathematical Modeling Reveals the Role of Hypoxia in the Promotion of Human Mesenchymal Stem Cell Long-Term Expansion
Source: Stem Cells Int. 2018 May 14;2018:9283432. doi: 10.1155/2018/9283432 (PMC5976908; doi:10.1155/2018/9283432)
Supplement: Supplementary Materials — The analysis results for the other dataset (with MSCs from a 78-year-old male donor) are presented in Table S1 and Figures S1–S3, corresponding to Table 1 and Figures 4 –6 in the main text. Table S2: model parameter values for normoxic and hypoxic conditions fitted from data in experiment A but with 10% nondividing cells at the beginning. Figure S4: model fitting results by setting T h = T n in experiment A as a comparison to Figure 4 in the main text. Figure S5: model fitting results by exchanging the net expansion rate r 1 in Table 1 as a contrast of Figure 4 in the main text. The detailed fitting procedures are also presented. [file 9283432.f1.pdf]

Supporting information for "Mathematical modeling reveals the role of hypoxia in promotion of human mesenchymal stem cell long-term expansion"

Table S1 Model parameter values for normoxic and hypoxic conditions fitted from another dataset in Experiment A, i.e., the one with MSCs collected from a 78-year-old male donor. r_1: net expansion rate; r_{20}: death rate of non-linear cells; L, k and T are the upper bound, the steepness and the midpoint time of the logistic state transition rate $r_{12}(t)$ respectively.

**Parameter**	**Normoxia**	**Hypoxia**
r_1 (day^{-1})	0.3790	0.3903
r_{20} (day^{-1})	0.0293	0.0537
L (day^{-1})	0.3943	0.4131
k	0.1482	0.1433
T (day)	48.9356	64.9611

Table S2 Model parameter values for normoxic and hypoxic conditions fitted from data of a female donor of age 56 (same as the main text) in Experiment A but with 10% non-dividing cells at the beginning, i.e., $x_2(0) = 0.1y(0)$, for comparison with Table 1. r_1: net expansion rate; r_{20}: death rate of non-linear cells; L, k and T are the upper bound, the steepness and the midpoint time of the logistic state transition rate $r_{12}(t)$ respectively. We can see that the result is almost the same as Table 1 in the main text.

**Parameter**	**Normoxia**	**Hypoxia**
r_1 (day^{-1})	0.3506	0.3528
r_{20} (day^{-1})	0.0136	0.0141
L (day^{-1})	0.3623	0.4094
k	0.3015	0.3565
T (day)	47.2027	72.4237

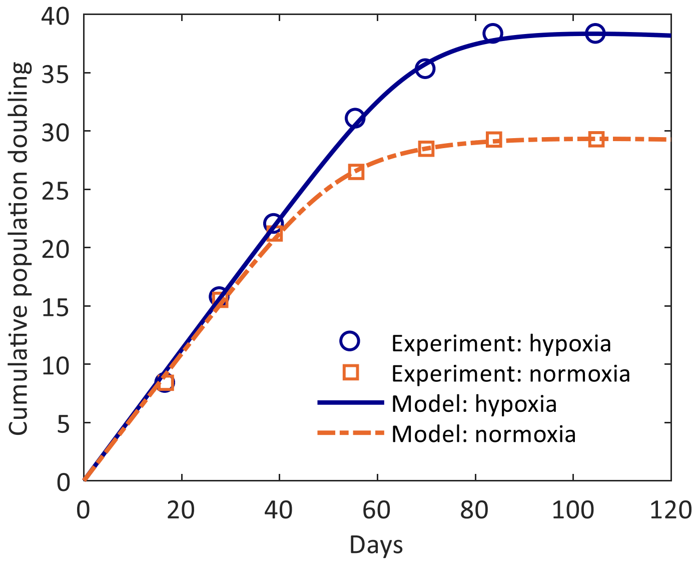

Figure S1 Experimental measurements and model-fitted population dynamics of MSCs at different oxygen tensions in Experiment A for another dataset (MSCs from a 78-year-old male donor). The cultured MSCs under study were originally obtained from a female donor of age 78

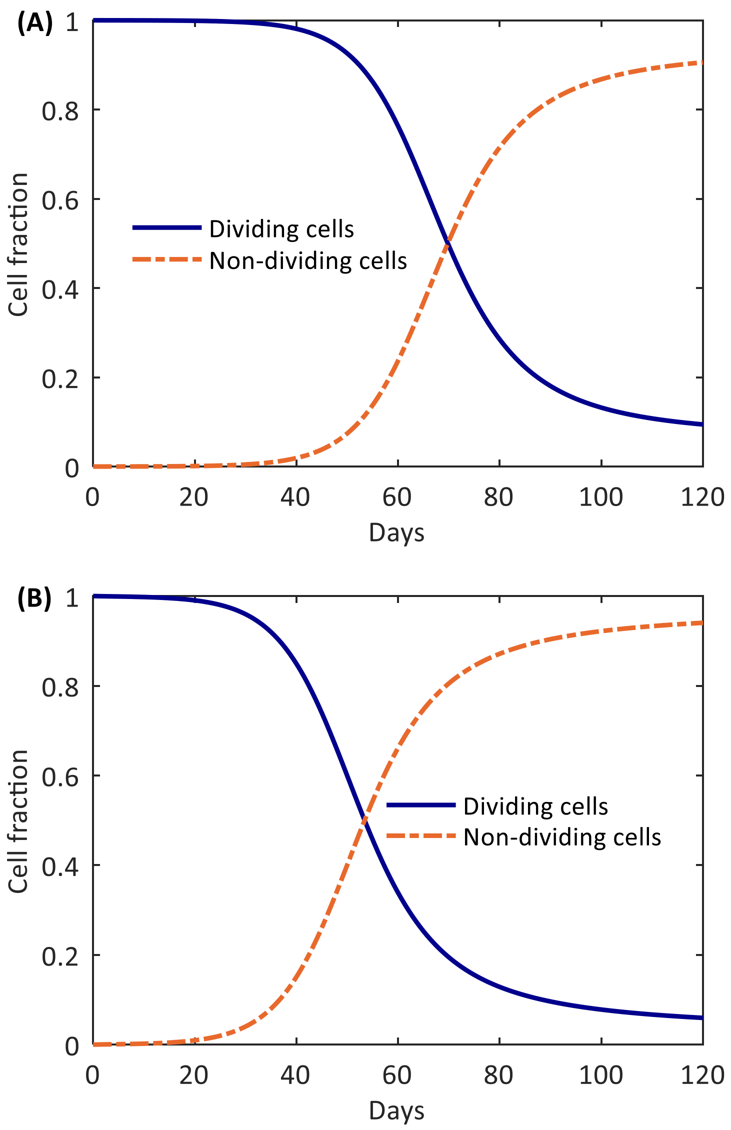

Figure S2 Simulation of the dividing and non-dividing cell fractions in the two oxygen environments of Experiment A for another dataset (from a 78-year-old male donor). (A) Hypoxia. (B) Normoxia. Initially (at day 0), it is assumed that all cells are dividing in both two conditions.

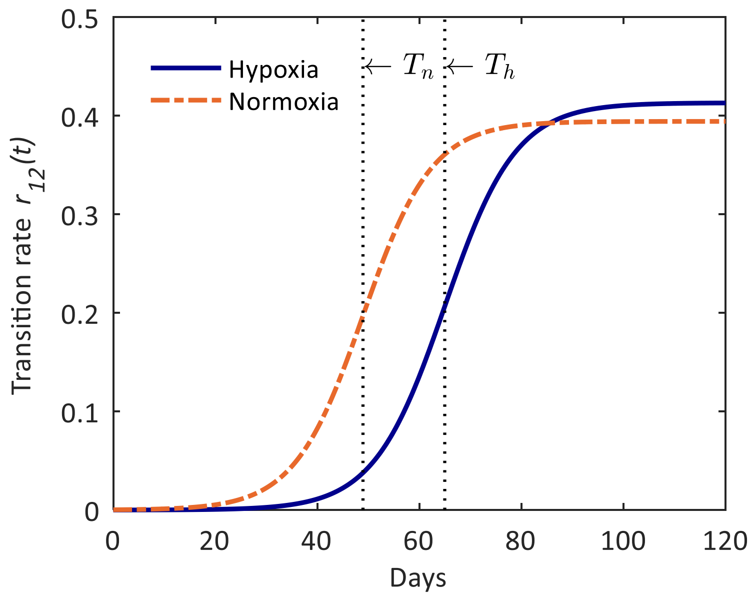

Figure S3 Comparison of the time-variant state transition rate $r_{12}(t)$ for MSCs cultured under hypoxia and normoxia in Experiment A for another dataset (from a 78-year-old male donor). The three parameters used to simulate the logistic function $r_{12}(t)$ defined in equation (2) can be found in Table S1, of which the midpoint time is annotated in the figure as T_n and T_h for normoxia and hypoxia respectively.

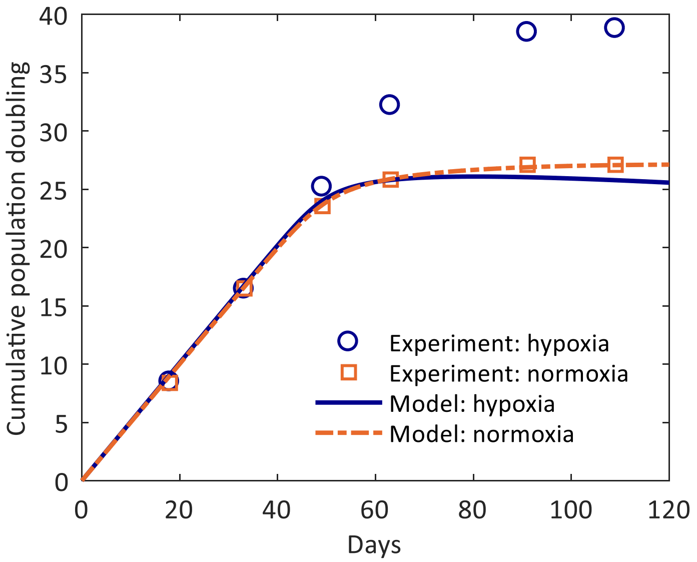

Figure S4 Model fitting results by setting $T_h = T_n$ in Experiment A as a comparison to Figure 4 in the main text. The other parameters for this simulation are listed in Table 1. The MSCs under investigation are collected from the 56-year-old female donor (data used in the main text Experiment A). As shown here, the slightly different L in the two conditions cannot fit the hypoxic data and thus cannot explain the considerable difference of cell yields in these two conditions. In other words, it is the parameter T that can explain the disparity of MSC expansion under the two oxygen conditions.

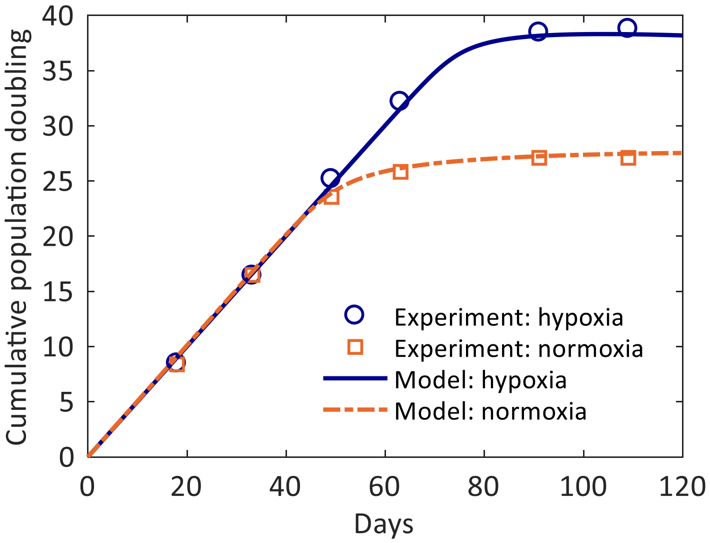

Figure S5 Model fitting results by exchanging the net expansion rate r_1 in Table 1 as a contrast of Figure 4 in the main text. As shown here, even if we assume the net expansion rate r_1 is slightly larger in the normoxic condition, there is a still a large gap between the final population size under the two conditions. Besides, the fitting results change quite little in comparison with Figure 4. Thus, we can conclude that the minute difference of the fitted r_1 value under two oxygen conditions plays just a negligible role in causing the significant difference of MSC expansion efficiency.

Implementation details of nonlinear regression in MATLAB

In this section, we present more details about how to solve the nonlinear regression problem (5) corresponding to Figure 3 in the main text, i.e., how to minimize $J(\boldsymbol{\theta})$. Once we choose an initial value $\boldsymbol{\theta}_0$ for the parameter vector, we can simply feed the data into mature optimization algorithms in MATLAB, such as *fminsearch*, *fmincon* or *lsqcurfit*. However, it is well known that the optimization of a nonlinear, nonconvex objective function may be sensitive to initial values due to the existence of multiple local optima. Therefore, in the following we mainly explain how to choose a *good* initial value for the parameter vector $\boldsymbol{\theta}$**.** In our study, since for each experiment there are only few data points, we simply use an exhaustive grid search method to determine the initial parameter values [1], detailed as follows.

Step 1: Determine a minimal parameter space, according to our prior knowledge or by observing the experimental data.

In equation (5) of the main text, the constraints for the parameters are derived from merely mathematical requirements. However, with some prior biological knowledge or simply by observing the experimental data, we can further narrow the parameter space.

For example, with the data points of Experiment A presented in Figure 4, it is easy to estimate the slope of the first stage, i.e., the exponential growth stage, to be around 0.5. According to equation (8), we know that the theoretical slope is $r_1 \log_2 e$ and thereby the value of r_1 should be less than 0.5, since $\log_2 e \cong 1.44 > 1$. As for the parameter r_{20}, which represents the death rate, it should be a small value according to our biological knowledge of MSC proliferation: we simply guess that $0 < r_{20} < 0.2$. It is also easy to determine a rough range for the parameter T, the midpoint time of the logistic function, by noticing that in Figure 4 the exponential growth doesn't stop until day 50 and the plateau stage is reached around day 80 for both conditions. Thus, we know approximately $50 < T < 80$. For the other two parameters, L and k, it is a little difficult to further narrow their range, and we preserve their upper bound listed in (5), i.e., $r_1 < L < 1$ and $0 < k < 1$.

With such a narrowed parameter space, it is easier to choose sensible starting-values for the optimization.

Step 2: With the parameter space determined in step 1, perform a coarse-grained grid search to try different initial values for the optimization routine.

The classical grid search method constructs a discrete grid based on the parameter space C composed of all parameters. Usually, the points are sampled equidistantly as the initial values to be tested for optimization. In our implementation, for this 1st-round coarse grid search, we pick 5 equidistant values in

the range of each parameter. Therefore, for the total 5 parameters, there are $5^5 = 3125$ trials made in total. That is, we have tested 3125 initial parameter values uniformly sampled from the parameter space, and for each initial value the optimization routine is executed and yields a minimizer $\widehat{\boldsymbol{\theta}} = \min_{\boldsymbol{\theta}} J(\boldsymbol{\theta})$, which depends on the initial values $\boldsymbol{\theta}_0$. Define the *best* minimizer in this round as the one leading to the minimum objective value: $\widehat{\boldsymbol{\theta}}^* = \min_{\widehat{\boldsymbol{\theta}}} J(\widehat{\boldsymbol{\theta}})$.

Step 3: Perform a fine-grained grid search around the best minimizer we obtained in step 2.

Since the number of grid points increases quickly with model dimension, in the above step 2 we only perform a coarse grid search with 5 samples for each parameter, and a further fine-grained grid search is made in this step. After we get the best minimizer $\widehat{\boldsymbol{\theta}}^*$ in step 2, we build a new grid with the neighboring points of the previously optimal solution $\widehat{\boldsymbol{\theta}}^*$. Suppose the optimal solution of a parameter p obtained in step 2 is p^* and the original range of this parameter is c_p, then a reduced range is specified to be $\overline{c}_p = [p^* - \frac{c_p}{10}, p^* + \frac{c_p}{10}]$, which should of course respect the fixed constraint in equation (5). This range reduction principle applies to all the parameters. Afterwards, another grid search routine like the one in step 2 is performed again in this reduced parameter space to further refine the optimal solutions.

The purpose of this fine-grained grid search step is to make it more likely that the global optimum is found. Even not, after the grid searches in step 2 and step 3, we have much confidence that a local minimum close enough to the global one can be obtained. Finally, the result we report is the best local minimizer found so far, $\boldsymbol{\theta}^*$, which leads to the minimum objective value among all trials in both steps.

In step 2 & 3, given an initial parameter vector, we used the nonlinear programming solver *fminsearch* in MATLAB to solve the nonlinear optimization problem. Particularly, the most important options are *MaxIter*=1000 (maximum number of iterations), *TolFun*=1e-4 (termination tolerance on the function value change) and *TolX*=1e-4 (termination tolerance on independent variable step). The optimization routine was actually finished in less than 700 iterations for most initial values. We have also tested other optimization options and other solvers such as *fmincon.* The obtained optimal solutions remains almost the same.

Remarks

1) It is known that the number of grid points increases exponentially with the dimension (number of parameters) of the optimization problem [1]. However, in our study, there are only five

parameters and the size of the dataset in each experiment is also very small. Thus, it is still practical to adopt the grid search method to attempt a good starting point for optimization.

2) To repeatedly run the optimization solver with different initial values (starting points), we can use a for-loop manually or resort to the *MultiStart* algorithm in MATLAB to execute these iterations automatically. Please refer to the online documentation for more details. https://www.mathworks.com/help/gads/multistart.html.
3) There is no technical reason to limit the solvers to be *fminsearch* or *fmincon*. Other solvers capable of nonlinear programming can also be used. Furthermore, global optimization solvers like genetic algorithm, differential evolution and particle swarm solvers are also good alternatives, though they may need a higher cost of computation. Interested readers may refer to [2] and [3].

Reference

[1] Schleer, F. (2015). Finding starting-values for the estimation of vector STAR models. *Econometrics*, *3*(1), 65-90.

[2] Price, Kenneth, Rainer M. Storn, and Jouni A. Lampinen. *Differential evolution: a practical approach to global optimization*. Springer Science & Business Media, 2006.

[3] Parsopoulos, K. E., & Vrahatis, M. N. (2002). Recent approaches to global optimization problems through particle swarm optimization. *Natural computing*, *1*(2-3), 235-306.
